# Current Trends and Potential Applications of Microbial Interactions for Human Welfare

**DOI:** 10.3389/fmicb.2018.01156

**Published:** 2018-06-01

**Authors:** Tiroyaone Shimane Tshikantwa, Muhammad Wajid Ullah, Feng He, Guang Yang

**Affiliations:** ^1^Department of Biomedical Engineering Huazhong University of Science and Technology, Wuhan, China; ^2^College of Life Sciences Huanggang Normal University, Huanggang, China

**Keywords:** microbial interactions, biofilms, quorum sensing, secondary metabolites, applications

## Abstract

For a long time, it was considered that interactions between microbes are only inhibitory in nature. However, latest developments in research have demonstrated that within our environment, several classes of microbes exist which produce different products upon interaction and thus embrace a wider scope of useful and potentially valuable aspects beyond simple antibiosis. Therefore, the current review explores different types of microbial interactions and describes the role of various physical, chemical, biological, and genetic factors regulating such interactions. It further explains the mechanism of action of biofilm formation and role of secondary metabolites regulating bacteria-fungi interaction. Special emphasis and focus is placed on microbial interactions which are important in medicine, food industry, agriculture, and environment. In short, this review reveals the recent contributions of microbial interaction for the benefit of mankind.

## Introduction

**Figure 1:**
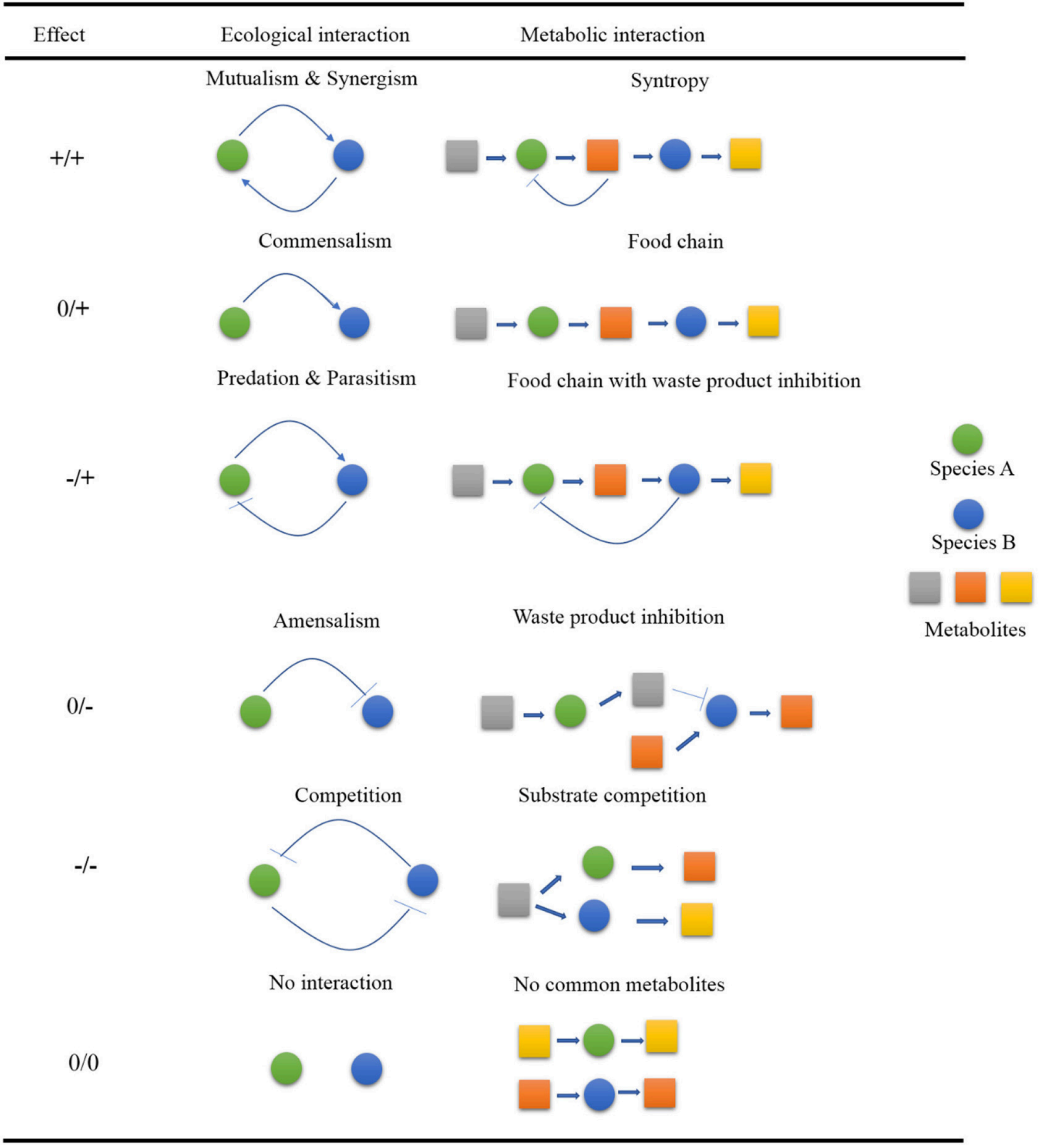
Summary of various interactive patterns of microorganisms in nature. In each of the partners interacting, there is a likelihood of positive (+), negative (–) or neutral outcomes. The metabolic networks can be used to model metabolic interactions.

Microorganisms like bacteria, fungi, algae, some parasites like protists and archaea, and viruses vary in shapes, size, and surface morphologies (Ullah et al., 2017b; Kiprono et al., 2018a,b; Shi et al., 2018) and often appear in nature to have formed some complex ecological interactive webs within the ecosystem rather than existing as single planktonic cells. These interactions among microorganisms can be between same species, with different species, or even among completely different genera and families. The interactive patterns within these webs are either positive (win), negative (loss), or neutral where there is no effect at all on the interacting species. The different win, loss, and neutral associations occurring between interacting partners provide a foundation for diverse forms of interactive patterns. A typical example of this scenario can be drawn from bacteria of differing taxa whose association can result in the development of a biofilm which may ultimately complex into making the individual members resistant to antibiotics. The resultant win–win relationship is termed as mutualism (Faust and Raes, 2012). In current day world, the different interactive patterns (Figure 1) noted could culminate into several diverse outputs; some of which have useful applications in various disciplines such as health interventions, food industry, agriculture, and environment, etc.

**Figure 2:**
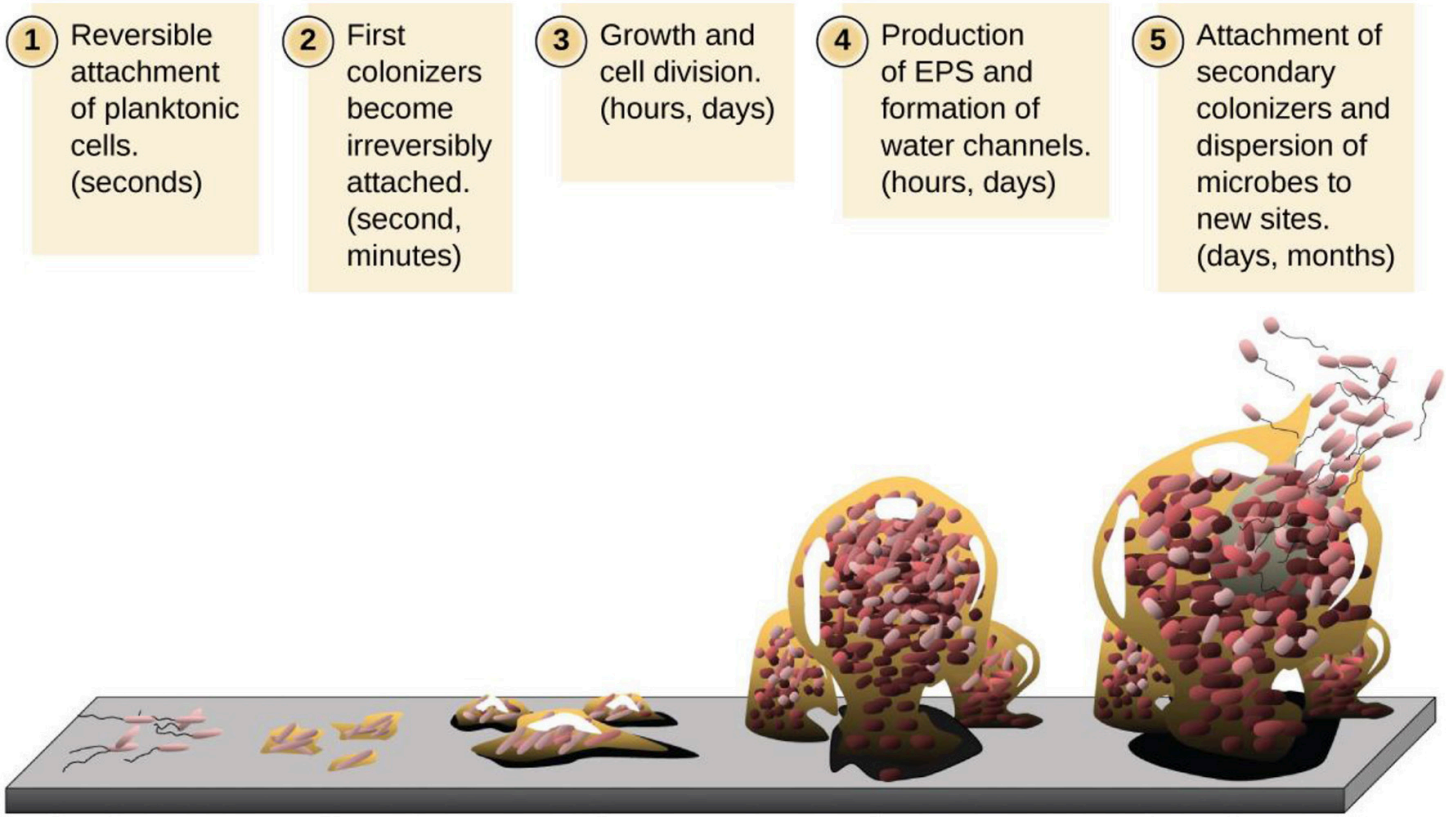
Description of various stages involved in the development of a biofilm. (1) Bacterial adhesion to the surface, (2) cell-to-cell adhesion, (3) attached cell monolayer, (4) maturation of a biofilm and formation of exopolymeric substance, and (5) detachment. (Image Credit: D. Davis).

Manipulating the microbial communities, on the other hand, is beneficial in treatment of waste water (Werner et al., 2011), food production (Mounier, 2008), and for disease prevention and treatment; for example, treatment of caries (Marsh, 1994), inflammatory bowel disease (Maloy and Powrie, 2011), and obesity (Ley et al., 2006; Blekhman et al., 2015). For instance, the gut ecosystem is mainly targeted for modeling purposes in case of inflammatory bowel disease and obesity. However, further efforts are required to develop models for gut microbiota (Faith et al., 2011). An enhanced understanding of the impact of microbial communities and better comprehension of the exact mechanism of operation of such communities has become a priority. The microbial communities influence life extensively in various disciplines; for example, human-associated microbiota impacts health, environmental microbes determine ecosystem sustainability, and microbe-driven industrial processes are expanding. In view of a wide range of applications, several means have been established to analyze and describe microbial communities (Zaccaria et al., 2017).

**Graphical Abstract:**
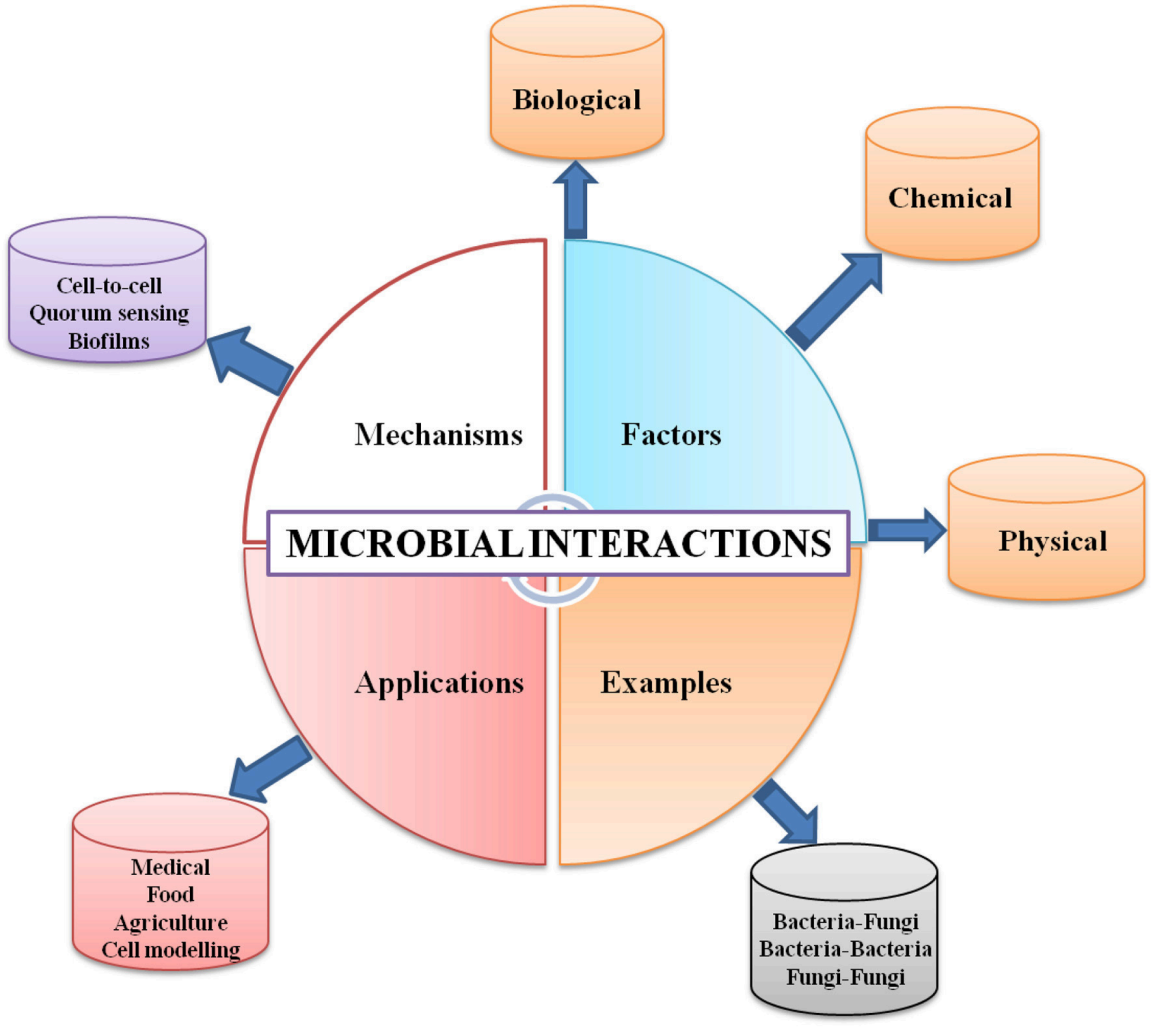
Overview of microbial interactions: examples; controlling factors controlling; mechanism of occurrence, and applications.

Microorganisms often modify their own phenotypes and processes of development such as biofilm formation or sporulation and tend to behave like factors of virulence in symbiotic and pathogenic interactions which involve additional members within a consortium. A scenario of unique relationships of mutualism exists whereby chemical synthesis capabilities ofsymbionts are used by the host organisms harboring them to prevent growth of any other potential competitors in order to maintain a certain unique lifestyle (Faust and Raes, 2012; Sheth and Taga, 2017). Consequently, complex networks of microbial ecology have resulted in clear understanding of basic biological processes and unearthed the noble virulence factors and developed various potential substances which can be used as drugs. However, a major challenge is to devise means through which safety of food is ensured and guaranteed through reduction or total elimination of contaminants from surfaces where food is handled (Tshikantwa et al., 2017). This elimination or reduction can be enhanced by preventing the attachment or destruction of bacterial cells assemblage to form biofilms. However, conditions favoring biofilm formation and ultimate attachment are unclear; hence most studies have been focused on unveiling the mechanism involved in biofilm formation. The management and prevention measures in biofilm formation are the major constrains, thus a comprehensive review will provide a gateway into the outlook of best possible management. Thus, the current review discusses various interactive patterns of microorganisms and their control mechanisms. Further, it reveals various useful mechanisms in view of providing a base to solve several life-threatening challenges of healthcare and medicine, agriculture, environment, food, and beverages.

## Classes of microbial interactions

### Positive interactions

#### Mutualism

It refers to an interactive form embracing all ecological associations and “cooperation” that describes mutualistic relationship between individual microbes as opposed to groups (Faust and Raes, 2012). The coexistence of interacting partners thus benefits both members accordingly. A common interest usually exists among the members of a community involved in such association. For example, bacteria commonly tend to synthesize certain metabolic factors which are ultimately released to the exterior of the cell across cell membrane. One such example of resultant metabolites produced during mutualistic microbial interactions is siderophores. These are iron-scavenging molecular products produced by several bacterial and fungal taxonomic groups. Because the availability of iron in natural environment is minimal due to its appearance in insoluble form (Fe-III), the mineral iron is therefore a major limiting factor during bacterial growth. Microbial cells therefore tend to cater for this limitation by synthesizing certain enzymes which are eventually released into the interstitial space where iron becomes readily accessible (West and Buckling, 2003). Iron is therefore sequestered by the siderophores, thus making it metabolically accessible for bacterial cells (Wang et al., 2004; West et al., 2006).

A typical example of mutual benefit among interacting individuals is observed during the fermentation of sourdough where a synergistic interaction occurs among yeasts like *Saccharomyces exiguous* or *Candida humilis* and lactic acid bacteria (LAB), especially *Lactobacillus sanfranciscensis* (Gobbetti et al., 1994; Gobbetti and Corsetti, 1997). The mechanism of action of this metabolic pathway is such that when maltose is released by the action of yeast amylase on starch metabolized by *L. sanfranciscensis*, the glucose component (maltose derivative) becomes an excretory product of *L. sanfranciscensis*. This results in provision of carbon through maltose-negative yeasts. As a result, the growth of *L. sanfranciscensis* is triggered by yeast as these increase the concentration of amino acids and peptides *via* protein degradation or accelerated autolysis (Gobbetti and Corsetti, 1997; De Vuyst and Neysens, 2005). Several natural products of certain bacteria and fungi act as rich sources of useful antibiotic drugs and drugs-like anticancer chemotherapeutics, immune-suppressants, cholesterol-reducing drugs, and anesthetics which are commonly used to curb certain medical conditions (Medema and Fischbach, 2015). A recent study revealed that certain bacterial species, for example *Staphylococcus epidermidis*, produces 6-N-hydroxyaminopurine (6-HAP) that impairs tumor growth by inhibiting DNA polymerase activity. The 6-HAP expressed selective antiproliferative function against transformed tumor cell lines, suppressed the induced growth, and *de novo* synthesis by UV exposure. This observation suggests that commensal skin bacteria has the potential to assist in defending host against neoplasia of skin (Nakatsuji et al., 2018).

#### Protocooperation (synergism)

Protocooperation is a class of interaction where participating partners benefit; however, the association is not obligatory and both populations can survive on their own. Nevertheless, such association provides a mutual benefit to the interacting partners. In general it is usually difficult to establish whether the relationship can be beneficial to both interacting organism (mutualistic), synergistic, or commensalistic. Some examples of synergistic interaction include: (1) formation of a product where none of the population can function on its own: for example, pathway completion (syntrophy), (2) affinity of microorganisms for each other as evidenced in usual occurrence of bacteria on algal surfaces related to chemotactic relations among them, (3) ability of some microorganisms to utilize toxic end products for their metabolism; (4) production of degradative enzymes, like those produced by *Arthrobacter* and *Streptomyces* (soil flora) which work together on organophosphate pesticide and degrade diazinon. This product is involved in the degradation of xenobiotics or recalcitrant compounds (Schink, 2002).

#### Commensalism

In commensalism, one interacting organism derives benefit from the association while the other partner remains unaffected. While this may occur in natural environment, it equally takes place in a number of fermentation processes involving foods by trophic interactions. Specifically, in Swiss-type cheeses, propionic acid bacteria make use of lactic acid produced by LAB which acts as a starter (Mounier et al., 2005). Similarly, in surface-ripened cheeses lactic acid is metabolized by yeasts, *Debaryomyces hansenii*, and *Geotrichum candidum* filamentous fungi (Mounier et al., 2005). The cheese surface is thereafter deacidified and allows excessive growth of aerobic bacteria like *Arthrobacter* species, *Brevibacterium linens, Corynebacterium ammoniagenes*, and *Staphylococci*. Herein, the aerobic bacteria are believed to be benefiting from the association; however, *D. hansenii* and *G. candidum* remain unaffected. It may however not be easy to confirm whether there is no effect as long as information on growth measurement and survival is not available.

### Negative interactions

#### Predation

In the ecosystem, organisms are interconnected and involved in several interactive patterns among themselves as well as with their immediate environment; such relationships are important for organisms to grow temporally. Once there is any perturbation from one group of organisms within an ecological niche, there is the possibility of subsequent effect on the size of a population in the entire ecosystem. This is demonstrated, most clearly, by the prey-predator relationships where predator (hunter) feeds on its prey (hunted). A typical example of this scenario in microbial world is the one that occurs between *Vampirococcus* and *Bdellovibrio* (Society for General Microbiology, 1999). In such unique interaction, the interacting partners equally hunt down and prey on other bacteria. The *Vampirococcus* continues to feed and keeps on using binary fission to grow until it ultimately exits its prey which thereafter remains only an empty shell (i.e., cell wall, cytoplasmic membrane, and some intracytoplasmic inclusions; Society for General Microbiology, 1999). The predatory mechanism potentially provides basis for controlling harmful microbial species and enhancing useful ones.

#### Parasitism

In parasitic type of microbial interaction, one participating organism benefits over the other. Bacteriophages are the commonly known examples of microbial parasitism in nature. Phages are reported to be prevalent in food fermentations, for example those that are used in the same equipment repeatedly. There is a likelihood failure and loss of production in industrial fermentations due to phage attack through inactivation of the dominant strains in fermenting cultures (Sturino and Klaenhammer, 2006). A significant increase in comprehension of phage biology and subsequent interactive associations with their harboring organisms has recently showed a marked increase. The bacteriophages of LAB such as *L. lactis* and *Streptococcus thermophilus* have been widely studied biologically (Brüssow, 2001; Sturino and Klaenhammer, 2006). The outcomes of such studies in genome sequencing have been remarkable as exemplified by at least seven phages specific for *S. thermophilus* which were previously mapped successfully (Sturino and Klaenhammer, 2006).

#### Amensalism

Amensalism is a type of an interspecies relationship where one organism has a negative effect on the counterpart which remaining unaffected itself (Sieuwerts et al., 2008). It is common occurrence during food fermentation because the key products of preliminary metabolism like alcohols and carboxylates are potent growth inhibitors of microorganisms which usually result in food spoilage (Lindgren and Dobrogosz, 1990; Caplice and Fitzgerald, 1999). A typical example of such interaction is revealed during LAB metabolism that is optimized to enhance quick production of acid instead of growth effectively (Teusink et al., 2007). Another such example is the production of bactericidal compounds like bacteriocins which are synthesized by several food-fermenting LAB and are useful in population dynamics of mixed-cultures. Under normal circumstances, bacteriocin-producing strains result in a strengthened immune system and confer protection to the host against any harmful effects. A unique group of bacteriocins (lantibiotics), derived from LAB and several other Gram-positive bacteria, are particularly important. For example, nisin is a common metabolic lantibiotic synthesized by *Lactococcus lactis* and is widely used in food preservation. The base of activity of these lantibiotic is the enhanced permeability of cytoplasmic membrane that results in its depolarization (Entian and de Vos, 1996; Hyde et al., 2006). Other potent bacteriocins include plantaricin and pediocin, which are widely distributed among *L. plantarum* and pediococci, respectively (Diep, 2006; Wiedemann et al., 2006). Exploitation of broad-spectrum bacteriocins has been done to inhibit the excessive growth of microorganisms responsible for contamination and pathogenesis (Loessner et al., 2003; Allende et al., 2007).

#### Competition

In competition type of microbial interaction, usually occurring during fermentation, there occurs a competition for energy sources and nutrients among the interacting microorganisms (Sieuwerts et al., 2008). This often occurs in environments with high concentrations of carbon. One of the interacting organisms gets outgrown by the other that rapidly takes up nutrients and transforms them into biomass. For example, nitrogen is often limiting in dairy fermentations and competition for the readily available amino acids and small peptides present in milk initially occurs among the organisms themselves. In subsequent fermentation stages, the interacting partners compete for the peptides released by the action of proteolytic enzyme. The microorganisms thereafter synthesize proteases, transport systems, and peptidases. In mixed dairy fermentations, the ability to make use of amino acids efficiently determines the growth rate and dynamic nature of population (Juillard et al., 1995, 1996).

### Unique microbial interactions

#### Bacteria-fungi interactions

Different forms of interactions across the crucial pathogenic microorganisms provide the basis for most medical conditions especially if these affect the immunocompromised individuals. It is therefore of utmost importance to ensure thorough comprehension of attachment mechanism and signaling taking place during fungi-bacteria interactions. This will ultimately lay the foundation for other advances in therapeutic strategies to hinder the possibility of microbial infections and occurrence of diseases caused by polymicrobial infections. Bacteria and fungi are often found together in different ecosystems especially in biofilms where these remain attached to solid surfaces and interact through different signaling processes. Despite the time-span of their co-existence, the research based on exploring bacteria-fungi inter-relationships especially in context of multiple infections, is still minimal. However, descriptions of many wide-ranging interactive relationships among the pathogenic fungi such as *Candida albicans* and different bacterial pathogens are increasing routinely. Coaggregation and subsequent attachment of *C. albicans* to microbiota in oral cavity which is important in inhabiting the oral cavity is a common example of benefiical mutualistic interactions. Contrary, the interaction between *C. albicans* and *Pseudomonas aeruginosa* demonstrates both competitive and antagonistic relationships. The interaction between *Staphylococcus aureus* and *C. albicans* is another example of intriguing interaction that occurs to be of synergistic nature yet not fully characterized (Shirtliff et al., 2009).

Presently, there is minimal information available about various types of interactions among different organisms that colonize humans despite their effect on them. This field however needs to be cautiously considered because of the complex nature of mixed type of infections. A clearer comprehension of mixed species infections (e.g., bacteria-fungi infections) in humans is also crucial in that the consequences of such infections can vary from those caused by individual mono-species (Peleg et al., 2010). As a result, application of alternative methods of treatment becomes more needful. Pathogens which are naturally opportunistic are often part of the multispecies infections. Consequently, immunocompromised individuals are more prone to such infections. *Rhizopus oryzae* accounts for 60–80% cases of all human mucormycosis (Ibrahim et al., 2008). Considering the fact that *Rhizopus fungus* was found associated with toxin-producing bacteria (endosymbiotic), it sounds most likely that *R. oryzae* may also be a haven for endosymbionts (Lackner and Hertweck, 2011). Identification of bacteria-associated fungi and bacteria-free fungi were carried out in laboratory screening of isolates (Partida-Martinez et al., 2007; Ibrahim et al., 2008). The impact of endosymbiotic bacteria on the outcomes of infections using fly and mouse models were investigated which did not show any apparent distinction in pathogenicity among the fungi harboring bacterial endosymbionts and the ones with no bacteria despite the fact that endo-bacteria giving rise to rhizoxin exhibited cytotoxicity (Ibrahim et al., 2008). A relationship of *Rhizopus* sp. And *Burkholderia* sp. exemplifies the neutral type of interaction during infection. However, it is yet to be explored in humans as to whether or not the same behavior can still be evidenced. A number of discoveries and descriptions have been made concerning the synergistic interactions among fungal and bacterial human pathogens. The yeast *Cryptococcus neoformans* and bacterium *Klebsiella aerogenes* coinfection is one of the mostly studied such interactions. The virulence factor of *C. neoformans* is found to be melanin (Frases et al., 2006). *C. neoformans* depends on exogenous substrate because yeast is unable to synthesize melanin on its own. Melanization by *C. neoformans* is achieved when *K. aerogenes* supplies dopamine (Frases et al., 2006). This pigmentation confers protection to the microorganisms against both environmental stress and human immune defense (Frases et al., 2006). The other enhancer of fungal virulence as a result of interspecies association is the biofilm formation by *C. albicans* and *Streptococcus gordonii* which coexist in oral cavity. *S. gordonii* was shown to promote the growth of hypha and formation of *C. albicans* biofilms (Bamford et al., 2009). In a study, Bamford et al. revealed that this interaction occurs through physical and chemical signals. The physical interaction occurs by way of adherence while interspecies signal molecule, autoinducer-2, performs a chemical signal role (Bamford et al., 2009). Additionally, *C. albicans* takes part in the antagonistic interactions between bacteria and fungi.

During *in vitro* interaction between *C. albicans* and *P. aeruginosa*, the hyphal filaments of *C. albicans* gets inhibited (Frey-Klett et al., 2011). The inhibitory activity is caused by a bacterial quorum-sensing molecule, 3-oxo-C12 homoserine lactone. Similarly, other bacteria like *Xanthomonas campestris, Burkholderia cenocepacia*, and *Streptococcus mutans* leads to production of farnesol derivatives which suppress the growth of hypha of *C. albicans*. The reciprocal effect of farnesol and 3-oxo-C12 homoserine lactone is believed to be due to a 12-carbon chain present within their chemical structures (Hogan et al., 2004). The reason for this is that the other molecules bear a similar chemical structure with different carbon chain lengths that do not cause similar signaling effects (Hogan et al., 2004). In most common forms of fungi-bacteria interactions (FBIs), bacterial peptidoglycans have been shown to enhance *C. albicans* hyphal growth rather than inhibiting it. In presence of *C. albicans*-derived farnesol, the bacterial quorum sensing activity modulates the expression of viral genes in *P. aeruginosa* (Currie et al., 1999; Morales et al., 2010). The reciprocal effect of 3-oxo-C12 homoserine lactone and farnesol is a natural FBI phenomenon that provide basis for advanced applications in biomedicine and possibility of generating new products. Future research aimed at optimizing the synthesis of new products, based on this type of FBI interaction, can yield novel discoveries.

The interspecies outcome of an infection ranging from promotion of virulence to interaction antagonism has been revealed through investigations of various bacteria-fungi interactions occurring in humans (Burns et al., 1999). Further research on molecular antagonistic interactions may however be required and thus might provide new treatment avenues or the previously unknown target points.

#### Action mechanism of 5MPCA

**Figure 3:**
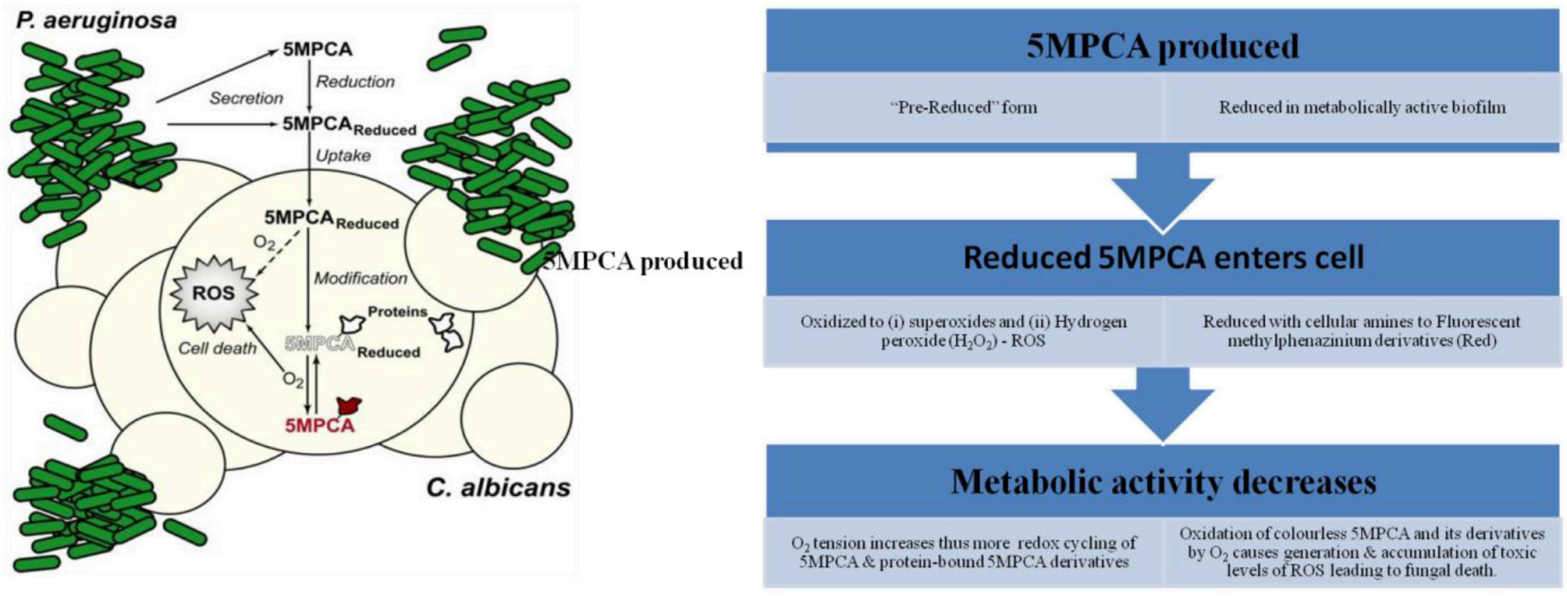
Proposed mechanism of action of 5-methyl-phenazinium-1-carboxylate (5MPCA). The figure has been modified from Morales et al. (2010).

Currently, there exists an antagonistic relationship between *C. albicans* and *P. aeruginosa*. The two pathogens have been often isolated from burn victims (Gupta et al., 2005) and cystic fibrosis patients (Hughes and Kim, 1973). *P. aeruginosa* gives rise to several resultant metabolites, for example pyocyanin (phenazine with antifungal activities), which either kill or inhibit *C. albicans* (Peleg et al., 2010). The 5-methyl-phenazine-1-carboxylic acid (5MPCA), precursor of pyocyanin, is reported to have high chemical potency over its final product (Gibson et al., 2009). The mechanism of antifungal activity of 5MPCA was proposed by Morales et al. (Figure 3) which demonstrates that redox-active 5MPCA synthesizes various reactive oxygen species (ROS) such as H_2_O_2_ and ${\text{O}}_{2}^{-}$. Additionally, 5MPCA reacts with amine moieties within proteins and results in impairment of vital cellular structures and enzymes. Derivatives of 5MPCA that have been covalently bound, maintain their reduction-oxidation activity as well as their ability to produce ROS. These two activities result in ultimate death of fungal cells (Morales et al., 2010).

### Regulation of microbial interactions

#### Physical regulation

##### Variation in salt concentrations has role in spoilage

Röling et al. reported that salt concentration has a significant effect on *baceman* spoilage. The yeast, which formed pellicle, grew at low salt concentrations. The same yeast brought about the presence of pellicle on the surface of liquid as they provided aerobic environment for coryneform bacterial growth. These bacteria utilized amino acids for metabolism and synthesis of fermentation products such as acetate and lactate, which led to increase in pH that ultimately resulted in spoilage of *baceman*. A similar growth pattern was shown by an aerobic *baceman* at low salt concentration (Röling et al., 1994b). These findings imply that the interplay of salt concentration and temperature greatly influence bacterial growth rate. This provides a possible base for modeling of microbial community to harness specific useful outcomes in terms of metabolites and other essential elements. The environment within which microbes interacts can be easily modeled by altering the physical factors such as temperature and pH to make them conducive for required outcomes.

##### Variation in temperature alters microbial enzymes activities

Temperature plays an important role in influencing the activities of interacting microbial enzymes. It was evidenced in formol nitrogen content and growth of microorganisms in Japanese *shoyu* (soy sauce) production where it was considered to be an important factor (Yokotsuka, 1986). Röling et al. proposed that a variation in temperature from 21° to 42°C during traditional *baceman* fermentation effects production of *kecap*. Consequently, three *baceroans* were generated from experimental *bungkil* and incubated at fixed temperatures of 24°, 30°, and 37°C (Röling et al., 1994b). The results indicated that higher temperature enhanced coryneform bacterial growth. Temperature appeared to have minimal effect on the final concentration of fermentation products; for example, the concentration of lactate was seemingly lower at 37°C as compared to 24° and 30°C; however, the concentration of ethanol was rather higher. The highest concentration of acetate was almost unchanged; however, declined slightly at 30° and 37°C after 14 days as a result of growth of pellicle-forming *Z. Rouxii*. In *baceman* maintained at 24°C, the pellicle-forming yeast constituted a small portion of entire yeast population, and there was no change in acetate concentration (Röling et al., 1994b). On the other hand, the population of *P. halophilus* seemed to play a significant role in this process as it was observed that the growth rate of *P*. *halophilus* increased at elevated temperature. The highest number of *P. halophilus* at 37°C reached its lowest of 2.9 × 107 CFU/mL (Röling et al., 1994b); however, it was comparable to traditionally prepared *baceman* from Libra where the numbers rapidly declined following *baceman* acidification. At 24°C and 30°C, highest populations of 5.0 × 10^8^ and 3.5 × 10^8^ CFU/mL of *P. halophilus* were attained, respectively, in comparison with those at 37°C and traditional Libra *baceman* (Röling et al., 1994b).

##### Concentration of dissolved oxygen is directly related to microbial growth

Oxygen is an essential element for growth of aerobic microbial cells. For example, production of cellulose by *Gluconacetobacter xylinum* halts when dissolved oxygen in growth medium vanishes (Ullah et al., 2015b, 2016, 2017a). At a higher pH within the range of 5.4–6.0, the availability of dissolved oxygen subsequent to the newly prepared *baceman* and high salt concentration, have shown the growth of coryneform bacteria. The coryneform bacteria growth led to oxygen depletion in *baceman*, which subsequently inhibited their growth progression. The disappearance of oxygen coupled with elevated pH due to inability of coryneform bacteria to produce acid, resulted in growth of salt-tolerant *P. halophiles* (Röling et al., 1994b). The acetate pH declined as its growth is associated with lactate production. This ensures the availability of dissolved oxygen to be a vital factor during the interaction of microorganisms within a community.

##### Microbial growth is ph specific

The pH plays an essential role in the ability of microorganisms to survive in a wide range of environmental niches. This conclusion was affirmed by a study by Röling et al. who revealed that variation in pH occurred during milk fermentation stage due to increased concentration of L-lactic acid were not sufficient to eliminate or inhibit the growth of either *Listeria* or *Staphylococcus* (Röling et al., 1994b). More significantly, pH values likely to influence survival of these pathogens; however, these changes occur very late during milk fermentation, if at all and are not likely to be achieved during normal fermented product manufacture. It is likely that variation in pH and L-lactic acid production over time in conjunction with other stages of processing may act in concert as critical control points.

##### Microbes require specific nutrients for growth and development

Specific nutrients are required for growth of different microorganisms to perform various metabolic activities. The quantity and type of specific nutrients vary greatly and mainly depend on type of microorganism itself. Sources of energy, nitrogen, minerals, vitamins, and water are examples of essential nutrients required by the microorganisms (Jay, 2000). These nutrients are available in foods at varying quantities; for example, meats are rich in protein content, minerals, lipids, and vitamins; while muscle foods have relatively lower amounts of carbohydrates. Consequently, such foods provide a suitable habitat for microbial interaction. Generally, foods of plant origin have higher concentrations of various carbohydrate groups and different protein quantities, minerals, and vitamins. The nutrients including carbohydrates, alcohols, and amino acids, etc. are utilized by microorganisms residing in food as their energy sources and metabolism. Most microorganisms tend to use simple sugars like glucose for their metabolism. On the other hand, other microorganisms are capable of metabolizing more complex carbohydrates like starch or cellulose foods of plant origin, or glycogen in muscle-related foods. Certain microbes still derive their energy from fats and amino acids as sources of nitrogen. Other examples of minerals necessary for growth of microorganisms include iron, sulfur, magnesium, manganese, phosphorus, calcium, and potassium. Gram-positive bacteria normally exhibit fastidious behavior with regard to their nutritional requirements, hence are unable to generate some nutrients necessary for growth; for example, food borne pathogen *S. aureus* utilizes amino acids, thiamine, and nicotinic acid for its development (Jay, 2000). Contrary, Gram negative bacteria possess the ability to generate and meet their most essential nutrients for survival from available carbohydrates, proteins, lipids, minerals, and vitamins which are present in a wide range of foods (Jay, 2000). *Salmonella enteritidis* is one of the pathogens with specific nutritional requirements. Growth of *S. enteritidis* is likely to be negatively influenced by the limited amount of iron. For example, albumen component of egg has antimicrobial agents compared to the yolk and thus is able to limit the availability of iron that in turn prevents the overgrowth of *S. enteritidis* to elevated levels. Humphrey illustrated that when iron was added to *S. enteritidis* inoculum in egg albumen, its growth was drastically enhanced (Humphrey, 1994). In general, the sequence of utilization of nutrients by microorganisms is such that first carbohydrates and amino acids are utilized followed by utilization of more complex nutrients. Food habitat is always complex because mixture of several microbes coexists in the same food concurrently. The availability of nutrients determines the growth rate of such microorganisms.

Relationship of direct proportionality exists between nutrient concentration and number of attached bacterial cells such that an increase in one variable gives rise to a similar increase in the other. However, biofilm development is supported by minimal concentrations of nutrients. Biofilms gain nutrients by re-concentrating the organic substances in minute amounts on surfaces by the extracellular polymer, utilizing the end products from their neighboring microorganisms and intermediary colonizers, and by pooling their biochemical resources using various enzymes to fragment food substrates. Since the biofilm matrix generally possesses negative charge, several nutrients (cations specifically) are often found attached to the biofilm surface. The negatively charged nutrients can be exchanged with ions on the surface. Individual cells within the biofilm are therefore provided with enough food rather than water available extracellularly (Cowan et al., 1991).

#### Molecular regulation of microbial interaction

The molecular level interaction among different microorganisms has led to a wide range of chemical diversity. Secondary metabolites with specific function in microorganisms play a pivotal role in mediating bacteria-fungi complex interactions in nature such as to ensure their survival in competitive environments. Consequently, several microorganisms have large biosynthetic potential as previously discovered in various whole-genome sequencing projects (Scherlach and Hertweck, 2009; Winter and Behnken, 2011). The varying requirements of changing environment often meet tightly regulating expression of biosynthetic gene clusters. Pure microbial cultures often harbor most natural product biosynthesis genes that seem to be dormant. The implication for this scenario is that several secondary metabolites remain untapped. However, the potential of bacteria-fungi interactions for drug-delivery has been largely exploited. This provided some limited information about secondary metabolism in cocultures while the approaches of polymicrobial cultures have been used extensively in order to have mass industrial production such as food and beverages (Scherlach and Hertweck, 2009). Previous researches have demonstrated the potential of mixed fermentations to influence the production of secondary metabolites. The core cultured organism impact was evaluated on the basis of antibiotic activity of culture extract or improved yield of a particular compound (Pettit, 2009). The research revealing newly discovered products derived from combination of pathways, biosynthesis, or activation received immense consideration. Cueto et al. developed a coculture of marine *Pestalotia* species with an unidentified antibiotic-resistant marine bacterium to ultimately elicit the benzophenone pestalone biosynthesis (Cueto et al., 2001). The resultant compound, which is only produced in mixed cultures, exhibited enhanced antibacterial potency toward methicillin-resistant *S. aureus* and vancomycin-resistant *Enterococcus faecium*. An actinomycete, *Salinispora arenicola*, was cultured in the same medium wit h a marine-derived *Emericella* species in a different study (Oh et al., 2007). Consequently, there was a 100-fold improved production of two new cyclic dipeptides, emericellamide A and B which led to elucidation of its structure. Moderate antimicrobial activity was recorded for emericellamide against *S. aureus* which is non-reactive to methicillin. A gene deletion protocol was used to discover emericellamide biosynthetic genes utilizing *Aspergillus nidulans* as a model organism where it paved the way for generation of innovative analogs (Chiang et al., 2008). The marine fungus, Libetella spp. was cocultured with a certain marine bacterium and four newly discovered diterpenoids, libertellenones A–D, were produced (Oh et al., 2005). A more systematic approach using microarray-based monitoring was utilized in gene expression cluster studies of cryptic biosynthesis in model fungus *A. nidulans* following the induction by interacting it with actinomycetes within the same ecological niche (Schroeckh et al., 2009). The findings further unveiled the complexity of bacteria-fungal interactions and gave further evidences in support to further study the microbial cross links in view of unearthing the discoveries yet to be made on the same interactions.

#### Cell-cell adhesions

Cell-cell adhesion is a mechanism through which microbial cellular interactive association occurs such that the cells become adherent to each other to surfaces or a metabolic substrate. The process is controlled by interactions taking place through cell surface molecules. This process results from the transmembrane glycoproteins action referred to as cell adhesion molecules such as selectins, integrins, and syndecans. In both prokaryotes and eukaryotes, the cell–cell communication *via* smaller signal molecules is critically important (Wang et al., 2004). In kingdom prokaryotae, several groups of cell–cell communication signals controlling different biological processes such as bioluminescence, plasmid transfer, virulence, and biofilm formation have been identified (Fuqua et al., 2001; Miller and Bassler, 2001; Chen et al., 2002). Cell–cell communication in bacteria plays a vital role in functional co-ordination within individuals of the same family in several biological activities that include expression of genes coding for virulence and biofilm formation (Wang et al., 2004).

#### Quorum sensing

**Figure 4:**
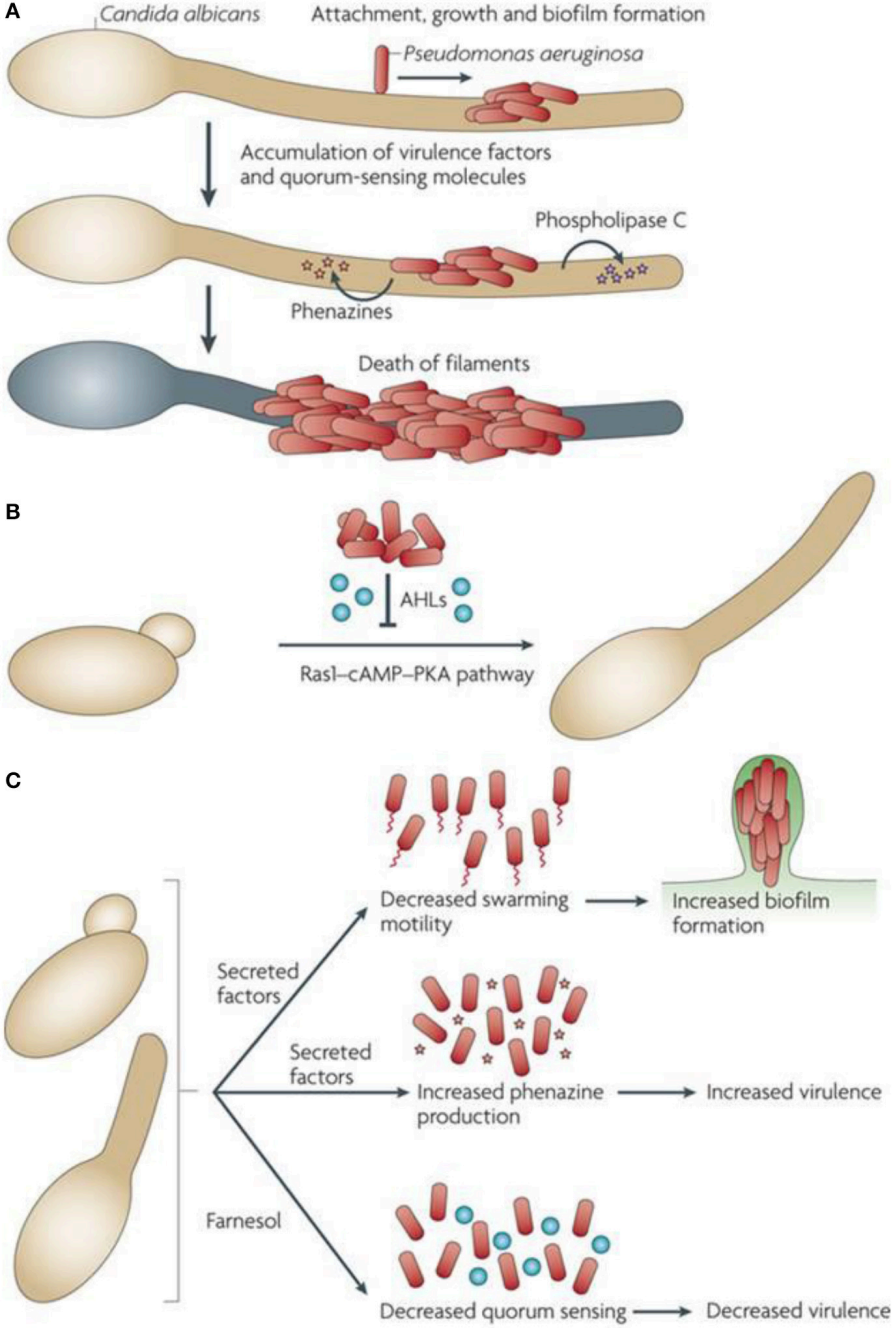
Interactive mechanism between *P. aeruginosa* and *C. albicans* at molecular level. The figure has been adapted from (Peleg et al., 2010). **(A)** Attachment of *P. aeruginosa* to the surface of *C. albicans* hyphae to form biofilms. Phospholipase C and phenazines are produced by *P. aeruginosa* leading to the death of fungi. **(B)** The quorum sensing molecules synthesized by *P. aeruginosa* and *C. albicans* in biofilm of a mixed-species get involved in autoregulation and communication among the species in biofilm itself (Hogan et al., 2004; Cugini et al., 2007; McAlester et al., 2008). The 3-oxo-C12-homoserine (an Acyl homoserine lactone – AHL) is produced by *P. aeruginosa* and inhibits the pathway of Ras1-cyclic AMP (c-AMP)-protein kinase A (PKA) for fungal growth in *C. albicans*. In this way the fungal filamentation is directly inhibited. Coincidentally, the coexistence of mixed species occurs because of elevated survival chances of yeast in the presence of *P. aeruginosa*. **(C)** The farnesol produced by *C. albicans* modulates the behavior of *P. aeruginosa* thereby altering the regulation of quorum-sensing. Virulence factors given rise by other uncharacterized *C. albicans* alter the biofilm formation and swimming movement of cells.

Several bacteria have shown ability to control their own cooperative activities and perform their specialized physiological functions through quorum sensing (QS) mechanism. In such situation, the bacterial cells exchange information among themselves by secreting, sensing, and responding to small diffusible signal molecules. These interactions add great advantages to the same organisms in host colonization, formation of biofilms, enhanced competition, and acclimatization to the altered environment. Most significantly, several QS-controlled activities are involved in virulence and possible pathogenic effects of bacteria (Figure 4). Consequently, true comprehension of molecular details for the mechanism of QS as well as their coordinated social activities may provide a breakthrough in managing emerging bacterial infections (Li and Tian, 2016).

During their growth phase, bacteria produce certain small mediating signaling molecules called auto-inducers which are involved in QS systems (Bouyahya et al., 2017). When these auto-inductors reach a certain concentration threshold, these interact with a transcriptional regulator and trigger specific expression of a group of genes. N-acyl homoserine lactone (AHL) produced by Gram-negative bacteria is a commonly studied intra-species auto-inducers. In this group of bacteria, QS genetic determinants are organized into a complex regulatory network including QS cascade and a spectrum of transcriptional and post-transcriptional regulators which affect the synthesis of AHL auto-inducer (Bouyahya et al., 2017). Over 70 known species of Gram-negative bacteria are known which use AHL as a signaling molecule (Sun et al., 2004; Lowery et al., 2008). Gram-positive bacteria, on the other hand, utilize a different auto-inducer, oligopeptide-based signaling with a two-component sensor. Apart from lactones, Gram-negative and Gram-positive bacteria can also use a common signaling molecule (borate furanosyl), known as auto-inducer-2 (IA-2) and (IA-3) (Bouyahya et al., 2017). QS plays a critical role in initial interactions occurring among microorganisms that provides an opening for extensive studies to utilize the action of QS molecules for controlling the microbial interactions and ultimately exploiting these for essential applications. This further provides an intervention mechanism in combating the existing insurmountable issue of antibiotic resistance (Adonizio et al., 2008). The emergence and reemergence of infectious bacterial diseases is lately countered by the most effective approach of targeting QS mediators (Hentzer et al., 2003). New anti-QS molecules are currently being considered as essential alternative measures to overcome major challenges presented by drug-resistant pathogens (antibiotics). To this end, several strategies have been proposed to interrupt and/or disrupt the bacterial QS system such as inhibition of signal generation and diffusion and inhibition of signal reception (Bouyahya et al., 2017).

The discovery of extensive use of QS systems in bacteria is critical in availing crucial information by researchers on bacterial multicellular behavior studies as opposed to emphasis on single cell biological processes. Studies on how QS plays a mechanical role in biofilms formation in bacteria, is however, still at its early stages of development. Determination of precise factors influencing biofilm formation and triggering QS and subsequent gene transfer still remains a major challenge in this field. In multi-species biofilms, the determination of functional consequences of QS equally becomes a challenge. This field is a key priority for research as it can potentially address any eminent challenges in the newly developing field of bacterial social behaviors. Undoubtedly, addressing the said challenges will contribute positively to the latest technology related to microbial behavior (Li and Tian, 2016).

#### Synthesis, structure, and composition of a biofilm

Over the time, microorganisms have proved that instead of existing as free living individual cells, these also appear attached to surfaces, structures, or as cooperative consortia referred to as biofilms (Douglas, 2003; Hentzer et al., 2003; Burmolle et al., 2006; Harriott and Noverr, 2011). In this state, these are rather resistant because these use this phenomenon to enhance their chances under enabling environmental conditions.

Biofilm formation occurs in a sequential manner (Figure 2) where the initial step involves either inorganic or organic molecules adsorbing on to the surface and creating a conditioning layer. In most cases, milk or meat derived proteins give rise to products that are essential components of this initial layer because these help in bacterial adhesion. For example, whey protein from milk preferentially increases adhesion of several milk-associated organisms in addition to increasing the bacterial adhesion in general (Kumar and Anand, 1998). The growth initiation of a bacterial biofilm generally occurs when individual cells initially attach to a surface (O'Toole and Kolter, 1998b). This process is greatly influenced by nutrients which increase the capacity of bacterial cells to attach to a surface (Watnick and Kolter, 2000). The subsequent stage in biofilm development is the attachment of organisms to the conditioning layer. This bacterial adhesion is controlled by several factors such as pH and temperature of the contact environment, fluid flow rate over the contact surface, nutrient availability, duration of bacteria-surface contact, bacterial growth phase, and extent of hydrophobicity of the surface (Kumar and Anand, 1998). An increase in hydrophobic status of a surface facilitates the bacterial adhesion to the surface; for example, stainless steel is a surface of high hydrophobicity capable of favoring adhesion for biofilms formation. *Bacillus* spores possess a hydrophobic surface due to their outer proteins, thus enhance the capability of adhesion on to the hydrophobic surfaces in comparison with viable reproductive cells. The spores, thus attach to stainless steel in higher levels than viable reproductive cells (Deibel and Schoeni, 2003). Examples of structures which enhance bacterial attachment to other surfaces include fimbriae, pilli, flagella, and exopolysaccharides (EPS) which connects bacteria and conditioning film (Kumar and Anand, 1998). The EPS possess electrostatic, covalent and hydrogen bonding, dipole associations, and hydrophobic relationship. Although at the initiation of microbial interaction, the EPS–bacteria bond is weak and can be easily destroyed by flowing water, it gets strengthened with time and the attachment become irreversible. When biofilm has reached this stage, cell detachment and subsequent removal may require a stronger action such as scrubbing or scraping. The non-swimming bacteria have reduced ability to form biofilms because these lack flagella. For instance, in most Gram-negative bacteria, about 1% of genome is devoted to flagella function. On the other hand, pilli provide twitching motility to bacteria through their (pilli) extension and retraction (O'Toole and Kolter, 1998a). This form of motility only occurs when cells are adherent to a surface and bacteria slide themselves across that surface. Consequently, the twitching movement is critical for synthesis of both microcolonies and spreading of biofilm communities (Ronney et al., 2004). The other step in synthesis of biofilms involves the growth of bacteria and its expansion to maturity. The biofilm maturation can occur within 24 h; however, its growth can proceed to millimeter sizes over several days (Deibel and Schoeni, 2003); this marks the complete formation of a bacterial biofilm unit. At this stage, the combined work of severe scrubbing and use of sanitizers is the best resort to remove biofilms; sanitizers alone are inadequate for such removal. At certain intervals, detachment and sloughing off of some bacterial cells within the biofilm consortia occurs. This is primarily as a result of reduced flow rate, the shearing effects of fluids coupled with chemicals within the fluid or altering the biofilm bacteria properties. The released bacteria are often transferred to new locations where formation of new biofilms is initiated (Kumar and Anand, 1998).

### Substrate utilization and metabolic needs for microbial interaction

In temperature-sensitive environment, the capacity of microbial growth of pathogens within an association of other microorganisms is determined by the interplay between internal and external factors in addition to the type of technology involved in the processing. For example, in food microbiology, several essential characteristics of microorganisms influencing the predominating species include the growth rate of individual microbial strains and shared interactive attributes among the combined populations (International Commission on Microbiological Specification for Foods [ICMSF], 1996).

The key determinants of length of microbial acclimatization period, generation time, and total yield of cells are genetically controlled. Accumulation of end products of metabolism has potential to retard the growth of some species (International Commission on Microbiological Specification for Foods [ICMSF], 1996; Ullah et al., 2014, 2015a). Due to the complexity of interplay between environmental factors and microorganisms, at any given time, food harbors its own microbial flora which continually associates with it (International Commission on Microbiological Specification for Foods [ICMSF], 1996). The availability of organisms that are metabolically active ensures that the occurrence of dominance in flora is a dynamically occurring process. These interactions can take either in an antagonistic or synergistic fashion depending on their growth-enhancing or inhibiting nature (International Commission on Microbiological Specification for Foods [ICMSF], 1996). Just as in the systems of foods, opposing actions often include competing for nutrients, for sites of attachment (space), unfavorable environmental changes, and a multiple of such factors (Jay, 2000). A similar scenario is equally observed when microorganisms interact in mixed culture within the environment; raw ground beef gives a typical example of this situation. The enterotoxin of *S. aureus* is totally unavailable, yet the bacterium itself is usually found in limited proportions in this product. The primary explanation for this is that *Pseudomonas-Acinetobacter-Moraxella* association that is often occurring in this food grows at elevated rate such that it outcompetes *Staphylococci* (International Commission on Microbiological Specification for Foods [ICMSF], 1996).

In case of extrinsic factors, antibiotic activity of gases at ambient and sub-ambient pressures on microbes are essential in foods (Loss and Hotchkiss, 2001). Microorganisms inhibit microbial life in two ways. First, their growth and proliferation can be inhibited by their direct toxic effect. Some of the gases such as carbon dioxide (CO_2_), ozone (O_3_), and oxygen (O_2_) poison some microorganisms. The key determinants of this inhibitory mechanism are chemical and physical properties of the gas itself and the association it has with both aqueous and lipid phases of food. Oxidizing radicals made by O_3_ and O_2_ are very toxic to the anaerobic bacteria; and depending on their concentration, these can have an inhibitory effect on organisms depending on oxygen for their survival. The extent of potency of carbon dioxide appears to be more expressed on obligate aerobes and when it is available at elevated proportions, it has the potential to prevent growth of other microorganisms. Another mechanism of inhibition is accomplished through modification of gaseous content, whose effect of inhibition is achieved indirectly through alteration of microbial ecology within the environment. The competitive environment is transformed when the atmosphere is also changed. In an environment where growth conditions disadvantage the multiplication of cells of one organism, the same situation may be beneficial to other microorgnaims, thus give it a nutritional advantage over its competitor. Based on native pathogenic microflora as well as their substrate, this situation may bring about beneficial or non-beneficial outcomes. An example of indirect microbial activity is the nitrogen replacement of oxygen (Loss and Hotchkiss, 2001). Generally, as the temperature decreases, the inhibitory effects of CO_2_ increase because solubility of CO_2_ increases at lower temperatures (Jay, 2000). The pH of food is lowered when CO_2_ is dissolved in it. As for nitrogen, since it is available in minute quantity in the atmosphere, it bears no antimicrobial properties completely. It is used separately or in combination with CO_2_ and as such has an indirect inhibitory effect on aerobic microorganisms (Loss and Hotchkiss, 2001).

## Possible applications of microbial interactions

### Medical applications

Bacterial–fungal interactions data on clinical relevance is currently limited. However, a number of studies have provided description on the relationship between bacteria and *Candida* spp. in several clinical specimens (Adair et al., 1999; Hermann et al., 1999; Klotz et al., 2007). However, it is unclear as to whether factors like systemic antibacterial therapy, host immune status, or exposure to nosocomial infections put a patient at risk of being colonized by both fungi and bacteria or not. Despite, infections by a combination of several species may have impacts compared to those infections caused by single species. In one study where ventilator-associated pneumonia (VAP) caused by *P. aeruginosa*, it was proposed that when respiratory tract is colonized by *Candida spp*., there was an increased reciprocal possibility of occurrence of pseudomonal VAP infections (Azoulay et al., 2006). This observation was also supported by other studies where individuals with tracheobronchial colonization by *Candida* spp. (i.e., those who received antifungal drug treatment) were least predisposed to pseudomonal VAP (Nseir et al., 2007). These findings were further validated by data from animal models. Utilization of human specimen from the past revealed that death due to bacteria or fungal infections (by *Candida* spp.) of bloodstream ranged from 10 to 40% (Gudlaugsson et al., 2003). However, a comparative analysis of single-species and mixed-species infections has been made by very few studies to date. One such study identified reduced survival chances for mixed bacteria–*Candida spp*. bloodstream infection than for an infection with *Candida spp*. alone (Dyess et al., 1985). As a result of rare possibility of prospective randomized human trials, the analysis of implications of mixed-species infections are limited (Jard et al., 2011). In this case, observational studies are made more complex owing to the fact that patients with multiple species infections pose a threat of other risk factors which are more characteristic of poor clinical outcome. For example, advanced capability of progression of illness or therapy can be adequately potent against one or both of the organisms involved in the infection. Moreover, a description of the mechanisms by which any variations in virulence occur at molecular level in a mixed microbial infection is difficult in human diseases studies (Jard et al., 2011).

### Food applications

A combined assemblage of microbes such as filamentous fungi, yeasts, and LAB plays a crucial role in preparing several fermented food products (Scherlach et al., 2013). This coculturing contributes immensely to the organoleptic characteristics of food and improves its quality variably. For example, it alters the aroma, flavor, and texture of food, and sometimes enhances the shelf life of fermented products as well (Dalié et al., 2010). A combination of mixed bacteria-fungus fermenting cultures is also utilized in several applications such as during production of alcoholic beverages like wine (Fleet, 2003) and beer (Rouse et al., 2008), dairy products (Viljoen, 2001) such as cheese and kefir (Lopitz-Otsoa et al., 2006), and sourdough (Gerez et al., 2009; Valerio et al., 2009). There are; however, very few investigations regarding the interactions between bacteria and fungi at cellular level during the fermentation process. Yeast cannot be used to enhance the utilization of bacterial starter as cultures because their cellular interaction mechanism has not yet been comprehensively elucidated (Viljoen, 2001). In contrast, a more detailed investigation in the antifungal metabolite-producing LAB molecular interaction and contaminating fungi in bakery products, cheese, and beer has been previously done (Rouse et al., 2008; Gerez et al., 2009; Valerio et al., 2009; Ryan et al., 2011). LAB produce antifungal compounds like organic acids (phenyl lactic, propionic, acetic, and lactic), hydrogen peroxide, cyclic dipeptides, hydroxy fatty acids, and reuterin, as well as uncharacterized proteinaceous and phenolic substances (Dalié et al., 2010). Moreover, the growth of contaminating bacteria *Listeria monocytogenes* in smear cheese can be inhibited by yeast (Goerges et al., 2006). Currently, extensive efforts have been devoted toward using the fermentation process to grow microorganisms that have inhibitory effects on bacteria and fungi responsible for causing adulteration, as need for additive-free foods has become increasingly important. Moreover, the risk of food spoilage by mycotoxin-producing molds or pathogenic bacteria can be minimized by using inhibitory starter cultures. This type of food poisoning reflects a reasonable risk to public health and poses threat to the economy. Biotransformation and biodegradation of mycotoxins by microorganisms is other latest research fields used to curb food poisoning (Jard et al., 2011). Food and other feed stuffs are often contaminated by such mycotoxins as aflatoxin, zearalenone, fumonisin, and trichothecene leading to toxic reactions upon subsequent uptake. These fungal toxins can be easily transformed or even degraded by several bacteria (Ibrahim et al., 2008). Useful as it is, the process of microbial degradation requires insightful critical analysis because not all reactions of biotransformation need mycotoxins detoxification. Moreover, an assessment is necessary to determine the stability of transformed product after upon humans and animals consumption (Scherlach et al., 2013). This can assist in determining its usefulness or possible long-term or short-term effects. Despite the fact that many bacteria investigated so far have indicated potential for degradation and transformation abilities, using biotransforming enzymes harnessed from the same organisms seem to exhibit higher potency compared to when live microorganisms were used for treatment (Jard et al., 2011).

The useful effects of microorganisms in a mixed culture (bacteria-fungi interactions) in food production, on the other hand, may as well cause contamination of food and as such greatly affect human health (Lackner and Hertweck, 2011; Table 1). This scenario is exemplified in Tempe bongkrek, a dish-derived from coconut and commonly available in Southeast Asia. This product is derived from coconut press cake fermentation using fungus *Rhizopus oligosporus* (Buckle and Kartadarma, 1990).

**Table 1:** Illustration of various physical, chemical, biological, and genetic factors and nutrients on microbial interactions.

**Factors**	**Parameters**	**Example of microbe type**	**Effect**	**References**
Physical	Salt concentration	Yeasts	Accumulation of spoilage yeasts in low salt concentrations.	Röling et al., 1994a
	Temperature	*P. halophilus*	Enhanced microbial activity at optimum temperature.	Röling et al., 1994a
	Dissolved Oxygen	Coryneform bacteria	Growth of coryneform bacteria favored by high O_2_ concentration.	Röling et al., 1994a
	pH	LAB	Favorable optimum acidic condition causes LAB to thrive more.	Coupe and Withers, 2012
Chemical		Fungi and bacteria	Vast chemical diversity generated interplay of various microbes at the molecular level.	Scherlach and Hertweck, 2009; Winter and Behnken, 2011
Biological	–	Different microorganisms	A microorganism can respond to environmental stimuli using metabolic exchange—the transfer of molecular factors, including small molecules and proteins.	Moree et al., 2012
Genetic	–	Different microorganisms	Provision of understanding of horizontal gene transfer, chemical signaling, pathogenesis, motility, chemotaxis, microbial viability and persistence.	Brehm-Stecher and Johnson, 2004
Nutrients	–	Different microorganisms	Different microbes have varying needs for water, a source of energy, nitrogen, vitamins, and minerals for their growth and maintenance of metabolic functions.	Mossel, 1995

### Agricultural applications

Employing knowledge about plant–microbe interactions benefits may help increase yield in production of food yet reducing environment and global biodiversity stress (Bloemberg and Lugtenberg, 2001). An essential ecological implication for function of soil which includes biogeochemical cycles is influenced by plants on the structure of a group of microorganisms and their role in the rhizosphere. This equally means that microorganisms residing in soil performs an important function in the health and productivity of plants (Bloemberg and Lugtenberg, 2001). The issue of essential function played by a microbial community that interact closely with plants to affect their health and biodiversity and productivity is currently greatly unexplored. Microorganisms offer some level of protection against plant diseases as an additional advantage to enhance nutrients supply to plants (Cavaliere et al., 2017). Particularly, different bacteria and fungi—especially of the genera *Pseudomonas, Bacillus*, and *Trichoderma*—produce several chemical products against other phytopathogenic fungi (Bloemberg and Lugtenberg, 2001; Walsh et al., 2001; Raaijmakers et al., 2002; Table 2). Biocontrol agents like those currently being used efficiently in this field; however, have not yet attained the required level of efficacy and consistency essential for large-scale commercialization. However, there are always chances for improvement and through continued developments microorganisms could be used as suitable choices in heavy fungicide regimens presently used in agriculture. Reducing the use of chemicals of this nature would be of direct benefit to the environment. It can also raise the appetite for ultimate consumers who crave for more natural products (Morrissey et al., 2004).

**Table 2:** Areas of possible applications of microbial interaction.

**Field**	**Example**	**Type of interaction**	**Product type**	**Application**	**References**
Medicine	Drug production	Bacteria-bacteria: *Penicilium notatum*	Penicillin	Treatment of diseases	Scherlach et al., 2013
		Bacteria-fungi *P. aeruginosa*-*C. albicans*	5MPCA	Antifungal agent	Marsh, 2003; Scherlach et al., 2013
Agriculture	Nutrient recycling	Bacteria-fungi: *S. cereviceae*–*R. etli*	Soil nutrients	Essential metabolite production	Helmholtz-zentrum and Res, 2016
Environment	Waste water treatment	Bacteria-bacteria: *Comamonas denitrificans* 110, *Brachymonas denitrificans* B79, *Aeromonas hydrophila* L6 and *Acinetobacter calcoaceticus* ATCC23055	Clean water	Waste water treatment	Andersson et al., 2011; Werner et al., 2011
Food and beverage production	Production of alcoholic beverages	Bacteria-fungi: *S. cereviceae; S. ovarum*	Alcohol	Production of alcoholic beverages	Fleet, 2003
	Food production	Fungi-fungi: *Streptococcus salivarius* subsp. *Thermophilus – Lactobacillus delbrueckii* subsp. *Bulgaricus*	Yogurt	Production of yogurt	Sieuwerts et al., 2008
	Production of cheese	Fungi – fungi: *Penicllium roqueforti-Rhizopus*	Surface-ripened cheese	Cheese production	Loessner et al., 2003
	Wine fermentation	Bacteria–Fungi: *Malolactic bacteria, yeast*	Wine	Wine production	Alexandre et al., 2004
Biotechnology	Vitamin synthesis	Bacteria-bacteria *Lactobacillus plantarum* SM39–*Propionibacterium fruendenreichii* DF13	Vitamin B12	Essential product synthesis	Hugenschmidt et al., 2010, 2011

Arbuscular mycorrhizal fungi (AMF) are commonly available forms in soil. These tend to establish direct physical link between soil and plant roots (Tiwari and Lata, 2018). Consequently, there is an elevated nutrient uptake as a result of increased surface area of plant roots-soil interface. This places the AMF at an important advantage to be directly involved in relieving the soil of heavy metal infestation which can be relayed to the plant host (Meharg, 2003). However, the ability of AMF to carry out this crucial function heavily rests on several factors such as the interacting species, types of metals and their subsequent availability, soil fertility, and conditions of plant growth (light intensity and density of roots; Pawlowska and Charvat, 2004). In addition, a tripartite interaction of pathogenic organisms, herbivore and plants as well, promises to be a useful relationship in enhancing crop yields (Willsey et al., 2017). This distinctively unique relationship relates viruses, fungi, and plants. This three-way symbiotic relationship has been found to be precursory for thermal tolerance (Márquez et al., 2007). It is further reported that a fungal endophyte and a tropical panic grass mutually grew at elevated temperatures of the soil. The explanation for this phenomena was traced back to the virus infecting fungi inside the grass. The findings were affirmed by testing the heat resistance of fungus that was treated by virus. Results revealed that the cured fungi could not withstand the temperatures as high as 65°C. Consequently, the heat tolerance was believed to be confered by the virus present in fungi within grass. This was because when fungi was reinfected wit virus, the heat-tolerance was restored. Based on the fact that virus provision of heat tolerance went beyond its native monocot host to its eudicot host, it was concluded that there must be some special pathways that characterize the mechanism of virus-fungi-plant interaction (Márquez et al., 2007).

Indepth biochemical studies of these pathways can provide further useful information to form the basis for usage of some virus-infected fungi in biocontrol mechanisms. Consequently, high crop yields will be rearlized, hence can results in a great breakthrough in agriculture especially in pest control.

## Significance of microbial interactions to the environment

Routine human activities such as farming, mineral exploration, and other forms of industrialization meant to sustain life have potential to adversely affect natural ecosystem (Tiwari and Lata, 2018). These effects come in the form of perturbations in soil, water, and air (Ul-Islam et al., 2016; Tiwari and Lata, 2018) which are the media of survival within the environment. A consorted interplay between plants and some species of microbes have been shown to assist in the control of accumulation of heavy metals in plants (Tiwari and Lata, 2018). The same interactions also have the ability to cause remarkable decline in the availability of harmful metals through a series of processes. Such interactive mechanisms are essential to prevent the subsequent damage to the soil texture. This is attributed to the effects of various elements in the soil (Panuccio et al., 2009; Hassan et al., 2017). The accumulation of elements in soil could have a negative effect on plant growth as it affects several physiological and molecular processes within the plants (Panuccio et al., 2009; Hassan et al., 2017). Examples of highly toxic metals include arsenic (As), cadmium (Cd), mercury (Hg), and lead (Pb), etc. These elements directly affect crop yields due to their elevated concentrations beyond certain thresholds (Xiong et al., 2014).

Within the environment, bacteria play an important role in treating the soil to redeem it of heavy metal contamination (Chen et al., 2015). The said bacteria accomplish this through three main processes namely: mobilization of metals themselves; transformation and detoxification (Tiwari and Lata, 2018). The specific mechanisms through which microorganisms accomplish these and other complex processes need to be thoroughly studied and highly explored. The most critical emphasis placed in the study of such mechanisms ought to be the focus on factors controlling them. Some of these factors can be traced to genetic and structural adaptation. These mechanisms can unearth new discoveries if their trends of occurrence are critically analyzed and studies overtime. Some fungal species like *Penicilium, Asoergillus, Muco*r, and *Trichoderma* possess special abilities to resist trauma due to heavy metals (Oladipo et al., 2018). This unique fungal property is related to the chemical structure of their cell wall. These can bind and opsonize metals because owing to the presence of various functional groups like phosphates, carboxyl, amines on their cell wall (Ullah et al., 2017b; Tiwari and Lata, 2018).

## Conclusions and future prospects

It is essential to understand the response of microorganisms to signals from plants in order to explore the usefulness of such interactions. As to whether or not, the future of biotechnological developments in agriculture depends on the technology of modification of genes or on traditional breeding, it should consider the benefits of plant–microbe associations. The “traditional” plant biotechnology sector centers around plant breeding and ultimately choosing the characteristics that are beneficial, and it least considers the ecology of plant–microbial association. However, the expression of desirable traits, such as resistance to diseases, or drought and salt tolerance, could also be directly influenced by interactions between certain variety of plant and its interacting microbial flora partner. A different scenario is also possible where certain varieties of plants may be involved in an interactive association with microorganisms of undesirable characteristics.

A clear interpretation of the genetic basis of interactions between plants and microorganisms considering the mechanism through which a certain plant may selectively identify its interacting partner within a pool of soil microbiota, gives way for “conditioning” of rhizosphere to promote more sustainable characteristics in the plant, which is effectively the basis of natural disease-suppressive soils. The biosynthetic gene cluster expression is often strictly controlled in order to meet the varying alterations of the environmental conditions. In pure cultures, biosynthetic genes coding for natural products normally remain silent hence several metabolic products are often not harvested. In this case, exploitation of mixed culture experiments in view of triggering such silent genes to ultimately harness the potential resultant metabolites becomes a priority of the current day research. Food fermentation in mixed cultures has an important economic advantage. The cultures consisting of LAB, yeast, and filamentous fungi are such that their performance is more than just the co-existence of functionalities of individual single-strain cells, but is primarily defined by substrate level interactions, the metabolites exchange, and growth enhancing or growth inhibiting factors.

Breakthrough following the latest developments in gene technology exposed new adventures of studying communities of microbes and interrelated networks more than the common models based on inferential descriptions. Additionally, studies whose basis was more on bringing an understanding on more basic ecological principles behind the success of evolutionary strategies utilized artificial laboratory strains and ecosystems. A more resourceful alternative technique with high practical relevance is thus provided by food fermentations. The in-depth study of current presence of advanced genomics and genetic tools have potential to allow the bringing together of mechanistic and evolutionary approaches to providing solutions to possible life challenges. Exposure to knowledge about various microbial interaction types and their usefulness provides a fertile platform for generation of well-organized synthetic microbiomes which can be applied in many settings; for example, solving crucial health related challenges and heavy metal remediation, etc. The natural metabolic activities of interacting cells are beneficially crucial in acting as “bio-engines” for essential metabolites such as volatile organic compounds (VOCs) and crucial metabolites.

## Author contributions

TT and MU designed the study, wrote the manuscript, and prepared the figures and tables. FH and GY critically reviewed and revised the contents and proofread the manuscript for typos and grammatical errors.

### Conflict of interest statement

The authors declare that the research was conducted in the absence of any commercial or financial relationships that could be construed as a potential conflict of interest.
